# Iranian consensus on use of vitamin D in patients with multiple sclerosis

**DOI:** 10.1186/s12883-016-0586-3

**Published:** 2016-05-21

**Authors:** Soodeh Razeghi Jahromi, Mohammad Ali Sahraian, Mansoureh Togha, Behnaz Sedighi, Vahid Shayegannejad, Alireza Nickseresht, Shahriar Nafissi, Niayesh Mohebbi, Nastran Majdinasab, Mohsen Foroughipour, Masoud Etemadifar, Nahid Beladi Moghadam, Hormoz Ayramlou, Fereshteh Ashtari, Shekoofe Alaie

**Affiliations:** MS Research Center, Neuroscience Institute, Tehran University of Medical Sciences, Tehran, Iran; Department of Clinical Nutrition and Dietetics, Faculty of Nutrition and Food Technology, Shahid Beheshti University of Medical Sciences, Tehran, Iran; Department of Neurology, Sina Hospital, Tehran University of Medical Sciences, Tehran, Iran; Headache Department, Iranian Center of Neurological Research, Neuroscience Institute, Tehran University of Medical Sciences, Tehran, Iran; Shafa Hospital, Medical Sciences University of Kerman, Kerman, Iran; Department of Neurology, Isfahan Neurosciences Research Center, Isfahan University of Medical Sciences, Isfahan, Iran; Department of Neurology, Namazi Hospital, Shiraz University of Medical Sciences, Shiraz, Iran; Department of Neurology, Shariati Hospital, Tehran University of Medical Sciences, Tehran, Iran; Department of Clinical Pharmacy, Faculty of Pharmacy, Tehran University of Medical Sciences, Tehran, Iran; Department of Neurology, Golestan Hospital, Ahvaz University of Medical Sciences, Ahvaz, Iran; Department of Neurology, Faculty of Medicine, Mashhad University of Medical Sciences, Mashhad, Iran; Department of Neurology, Azahra Hospital, Isfahan University of Medical Sciences, Isfahan, Iran; Department of Neurology, Imam Hossein Hospital, Beheshti University of Medical Sciences, Tehran, Iran; Department of Neurology, Neuroscience Research Center, Tabriz University of Medical Sciences, Tabriz, Iran

**Keywords:** Multiple sclerosis, Vitamin D, Etiology, Pathogenesis, Treatment, Assessment

## Abstract

**Background:**

Accumulating evidences from experimental, epidemiologic and clinical studies support the potential linkage between poor vitamin D status and the risk of developing Multiple Sclerosis (MS), as well as, an adverse disease course. However, the results of the trials on the clinical outcomes of vitamin D supplementation in MS patients are less consistent which brought many discrepancies in routine practice. In this article we presented a summary of a symposium on vitamin D and MS. In this symposium we aim to review the current data about the relationship between vitamin D and MS, and suggest management guides for practicing neurologists.

**Discussion:**

Generally, supplementation seems to be reasonable for all MS and clinically isolated syndrome (Rinaldi et al., Toxins 7:129–37, 2015) patients with serum 25(OH)D level below 40 ng/ml. In patients with vitamin D insufficiency or deficiency, a large replacing dose (e.g. 50,000 IU capsules of D per week for 8–12 week) is recommended. Panel also suggested: the checking of the serum vitamin D, and calcium level, as well as, patients’ compliance after the initial phase; a maintenance treatment of 1500–2000 IU daily or equivalent intermittent (weekly, biweekly or monthly) Dose, considering the patient’s compliance; routine check of serum vitamin D level at least two times a year especially at the beginning of spring and autumn; Serum vitamin D evaluation for first degree relatives of MS patients at high risk age and supplementation in case of insufficiency (25(OH)D less than 40 ng/ml); correction of vitamin D deficiency and insufficiency before pregnancy, as well as, a daily dose of 1500–2000 IU or equivalent biweekly intake in 2nd and 3rd trimesters; stopping supplementation if 25(OH)D serum level exceeds 100 ng/ml.

**Summary:**

Although the results of high power studies are not available, correcting vitamin D status seems plausible in all MS and CIS patients. Maintaining the serum 25(OH)D level between 40 and 100 ng/ml is not known to exert adverse effect. More ever, it might be associated with lower disease activity.

## Background

Vitamin D plays important roles beyond calcium hemostasis and bone health, including cell differentiation, proliferation and growth in the muscle, skin, parathyroid gland, pancreas and immune system. It influences the onset of diverse conditions such as cardiovascular and autoimmune disease. Sun shine is the necessary source of vitamin D [1]. We generally expose less than 5 % of our skin to sun shine and use Ultraviolet B (UVB)-protecting sun screen. Living in northern latitudes and industrial cities aggravate the situation. Only a relatively small number of foods contain substantial amounts of vitamin D, which cannot provide us with sufficient amount of vitamin D [2].

The probebility that vitamin D deficiency might be a risk factor for multiple sclerosis (MS) was suggested about 30 years ago. The role of vitamin D on etiology, pathophysiology and clinical course of MS has become a focus of intense research and has raised lots of questions and controversies among the physicians working with MS patients. Despite many investigations on this issue the optimal range of serum vitamin D and the proper dose of supplement are still unknown. Regular use of vitamin D has been considered by many health care professionals as well as MS patients who have received some information about the disease. Routine use of vitamin D in MS patients, their first degree relatives and people at risk of MS is still a debate and varies among different countries and even between practicing neurologists working at the same area. Consensus guidelines which answer some important questions on this topic according to the available evidences or experts’ opinions will help in preventing confusions and diversity of ideas during routine clinical practice. This paper resulted from 2 days discussion of a group of 14 experts including neurologists, a nutritionist and a clinical pharmacist who reviewed the current evidences about the relationship between vitamin D status and MS and tried to answer the most important questions of practicing physicians, regarding this subject. Information regarding vitamin D and MS (823 entries) were obtained from the pubmed database and the Cochrane database of systemic reviews. Relevant reports were considered from the initiation of the mentioned databases to April 2015.

### Review of current evidences

The link between vitamin D and multiple sclerosis was initially proposed after the discovery of higher disease prevalence in northern latitude that obtain lower amount of sunlight and consistently have lower vitamin D synthesis.

In-vitro and in-vivo studies have promoted the hypothesis of vitamin D and MS linkage. In-vitro studies showed that 1,25(OH)_2_D shifts CD4 T-cells and MHC class II molecules to a more anti-inflammatory profile. 1,25(OH)_2_D inhibits CD4 T-cells from developing to Th1 cytokine profile (TNF-α and IFN-γ). Also it promotes the expression of T-reg cells (secreting TGF-β and IL-10) and Th-2 (producing IL-13, IL-5, IL-4) [3].

Protective and/or therapeutic role of vitamin D was also reported in Experimental autoimmune encephalomyelitis (EAE)-the animal model of MS. Most of these studies started to feed mice with active form of vitamin D before EAE induction. Thus they mostly assessed the mechanism of protective role of vitamin D in EAE.

The proposed underling mechanisms for this relationship are: Inducing inflammatory cells apoptosis [4] i.e. CD4+ T-cells [5], suppressing immune cell infiltration into the CNS [4], i.e. CD 11b + monocytes [5], decreasing Inducible Nitric Oxide Synthase [4, 5], as well as, inhibiting proinflammatory cytokine secretion including IL-12 and IFN-γ [6, 7].

However, a number of studies proposed that vitamin D protective role might depend on its interaction with other factors such as inflammatory and anti-inflammatory cytokines. Inadequate IFN-γ may undermine vitamin D mediated inhibition of demyelinating disease. MS risk might increase in persons with insufficient IFN-γ expression, despite high sun exposure due to low Vitamin D Receptor gene expression and a high T-helper 1 and T-helper 17 cells in the CNS [8]. Vitamin D was failed to ameliorate EAE in mice with IL-10 and IL-10 receptor disrupted genes [9].

Strikingly, at least three studies revealed that serum vitamin D deficiency itself impairs the development of EAE. Deluca et al., maintained mice on vitamin D-deficient diet for two generations. EAE onset was postponed and the severity of EAE was ameliorated in D-deficient mice compared to controls [10]. Fermendes et al. reported that the offspring of vitamin D-deficient mice developed delayed and milder EAE. The over expression of TNF and osteopontin, as well as, the under expression of IFN were noted in the cerebellum and spinal cord of the second generation of vitamin D-deficient mice [11]. In Wang et al. study, VDR-knocks out mice with either sufficient or insufficient vitamin D intake, showed delayed onset and reduced severity of EAE. Wang et al. concluded that protective role of vitamin D against EAE is related to the accompanied hypercalcemia. Also, hypercalcemia induced by parathyroid hormones attenuated EAE in female mice [12].

As the absence of VDR and vitamin D deficiency inhibits the development of EAE, it is clear that the lack of the vitamin D signaling could not be a risk factor in EAE. More ever, a number of studies examined the role of UV light independent of vitamin D in protecting against EAE. The results revealed that UV itself attenuates EAE severity [13]. The observed discrepancy in the results of the animal studies raised the following questions: Does vitamin D alone has protective role against EAE? What is the cause and effect relationship between vitamin D-insufficiency and EAE?

### Human studies

#### Epidemiologic studies

Epidemiologic studies strongly support the hypothesis of vitamin D insufficiency as a risk factor for MS. According to the Nurses’ Health study II cohort results, MS risk was lower in women whose mothers had higher predicted 25(OH)D during pregnancy [14]. Antico et al. reviewed the studies on vitamin D and MS risk. They concluded that low vitamin D level especially levels lower than 10 ng/ml could aggravate autoimmune disease such as MS [15]. Some studies highlighted the needs for considering ethnic/racial differences in MS susceptibility. For example, the results of the MS sun shine study which performed by Langer-Gould et al., reported that the vitamin D hypovitaminosis is not a MS risk factor in Hispanics and blacks [16].

Studies also assessed the timing of vitamin D insufficiency on MS susceptibility. Two studies reported the higher risk reduction in people who have vitamin D serum levels of ≥30 ng/ml when they are under 26.4 and 20 years old [17, 18].

#### Cross-sectional studies

A literature review by Simon et al. distilled the following results from the accumulating evidences: (1) Controversy in the serum level of vitamin D MS patient have typically lower serum 25(OH)D levels. 25(OH)D level tends to be stable before the onset of symptoms, but reduced thereafter. This might reflect decreased outside activity and sun shine avoidance rather than the effect of vitamin D-deficiency on the risk of MS [19]. On the other hand, a number of studies reported a higher 25(OH)D level in MS patients. To date with the great attention to the possible therapeutic effect of vitamin D, many of the MS patients receive vitamin D supplement. A study reported that 7 % of MS patients were taking vitamin D before MS diagnosis in comparison to 66 % after diagnosis [19].

(2) Ddiscrepancy in the results of studies that assessed the effect of vitamin D insufficiency on disease severity.

A study reported no significant correlation between D level and Expanded disability status scale (EDSS) or multiple sclerosis severity scale (MSSS) but another study [19] observed significant relationship between higher 25(OH)D level and decreased MSSS [19]. In addition to Simon et al. report, Thouvenot et al. reported that in relapsing-remitting multiple sclerosis (RRMS) patients, the correlation between EDSS and vitamin D level was only significant in patients with EDSS < 4 [20].

Simon et al., concluded that the cross-sectional design does not allow deduction regarding temporality [19].

#### Clinical trials

Vitamin D supplementation could have several immunological effects on MS patients. For example, 10,400 IU vitamin D/days decreased IL-17 producing CD4+ Tem cell and CD4+ T cells in MS patients [21]. However, the results of the studies on the clinical/neurological outcome of vitamin D supplementation are less consistent.

In 2013 James et al. published a meta-analysis on vitamin D supplementation in MS patients. Five studies with a total of 129 vitamin D treated patients and 125 controls (90 placebo, 35 low dose of vitamin D) were enrolled (Table 1) [22]. No significant relationship was observed between high-dose vitamin D treatment and relapse risk. The authors concluded that the clinical trials were failed to provide satisfying evidence in favor of the therapeutic effect of vitamin D [22].

**Table 1:** Clinical trials on vitamin D for MS treatment

First author, date	Method	Participants	Intervention	Neurological/clinical measures	Neurological/clinical result
Burton, 2010 [58]	open-label randomized prospective controlled 52-week trial	MS patients 18–55 years mean EDSS: 1.34 Treatment group/control (n): 24/23	-Treatment: Increasing vitamin D3 dose up to 40,000 IU/d for 28 weeks, followed by 10,000 IU/d for 12 weeks, downtitrated to 0 IU/day plus 1200 mg calcium/d throughout the study -Control: = < 4000 IU vitamin D3/day and calcium if needed	EDSS, serum calcium level	No significant difference in relapse rate
Kampman, 2012 [59]	Randomized double-blind controlled 96-week trial	MS patients 18–55 years EDSS ≤ 4.5 Treatment group/control (n): 35/33	-Treatment: 20,000 IU vitamin D3/week, plus 500 mg calcium/day -Control:500 mg calcium/day	annual relapse rate, MSFC, EDSS, fatigue and grip strength	No significant difference in annual relapse rate, MSFC, EDSS, fatigue and grip strength
Shaygannejad, 2012 [60]	Randomized double-blind controlled 12-month trial	RRMS 15–60 years EDSS ≤ 6 Serum 25(OH)D > 40 ng/ml; and willing to continue vitamin D supplementation Treatment group/control (n): 25/25	-Treatment: 0.25 mcg calcitriol/day increased to 0.5 mcg/d after 2 weeks -Control: placebo	Relapse rate and EDSS	No significant difference in relapse rate and EDSS
Soilu-Hänninen, 2005 [61]	Randomized double-blind controlled 1-year trial	RRMS 18–55 years EDSS ≤ 5 Treatment group/control (n):34/32	-Treatment: 20,000 IU vitamin D3/week -Control: placebo (with interferon β-1b use)	EDSS, relapse rate, timed 10 foot tandem walk test, timed 25 foot walk test, brain MRI	Significant reduction in the number of T1 enhancing lesions and EDSS in treatment group No significant reduction in relapse rate
Stein, 2011 [62]	Randomized double-blind controlled 24-months trial	RRMS >18 years Treatment group/control (n): 11/12	Treatment:6000 IU vitamin D2 twice daily +1000 IU vitaminD2 daily Control:1000 IU vitamin D2 daily + placebo	MRI, relapse rate, EDSS	No significant difference in MRI findings. Follow-up EDSS was higher following high dose D2 (after adjusting for baseline EDSS), relapse rate was significantly higher in high dose group
Ongoing trials					
Clinicaltrials.gov identification number	Duration	Estimated enrollment	Intervention		
NCT01198132	96 weeks	250	Treatment: 100,000 IU vitamin D3/month + 3 rebif/week Control: 3 rebif/week		
NCT01490502	104 weeks	172	Treatment: 5000 IU vitamin D3/day + Copaxone Control: 600 IU vitamin D3/day + Copaxone		
NCT01024777	26 weeks	40	Treatment: 10,000 IU vitamin D3/day Control: 400 IU vitamin D3/day		
NCT01285401	96 weeks	358	Treatment: 6670 IU vitamin D3/day for 4 weeks, 14,007 IU vitamin D3/day for the following 92 weeks + 3 Rebif /week Control: 3 Rebif /week		
NCT01440062	78 weeks	80	Treatment: 20,400 IU vitamin D3 on alternate day + Interferon ß-1b Control: 400 IU vitamin D3 on alternate day + Interferon ß-1b		

The failure of clinical trials in reaching credible results might be due to the effect of factors that influence vitamin D function in the body (e.g. vitamin D Bounding Protein-DBP). Vitamin D circulates mostly in DBP-bounded form. Recent evidences found a DBP-dependent transport mechanism that facilitates the access of DBP-bound vitamin D to target organs. Of note DBP could inhibit the action of 25(OH)_2_D3 and 1,25(OH)_2_D3 on Antigen Presenting Cells (APCs). Also DBP reported to suppress the conversion of 25(OH)_2_D3 into 1,25(OH)_2_D3 by T Cells. One study reported the higher plasma level of DBP in MS patients compared to controls [1].

Another susceptible factor which could influence vitamin D function is vitamin D receptor (VDR). Results of a systemic review on VDR polymorphism showed that AA ApaI and FF FokI genotype could be significant MS risk factors. Another possible risk factor is TaqI. However, the relationship between TaqI polymorphism and MS susceptibility is highly affected by sample characteristics [23].

Briefly, the clinical trials failed to reach consistent results regarding the prevalence of hypovitaminosis D and the therapeutic effect of supplementation [22]. In MS patients, differences observed in factors which mediate vitamin D function such as higher plasma DBP level compared to healthy individuals [1] and VDR polymorphism [23] add more complexity to the question about vitamin D supplementation. In the presence of the mentioned discrepancy and complexity, results of an experts’ meeting might provide clinicians with useful information, till the release of the results of the ongoing large clinical trials (Table 1).

### Recommendations of the expert panel

Most frequently asked questions about vitamin D supplementation were discussed. The recommendations were scored using an agreement index ranging from 1 (not agree at all) to 5 (fully agree). The mean score is presented.

### Who should be tested for serum 25(OH)D level and what is the optimal range of vitamin D?

Although there is inconstancy in the results of cross-sectional studies that compare vitamin D status in MS patients and healthy subjects, the high prevalence of vitamin D deficiency in general population including MS patients is predominantly accepted. Thus, there was a general consensus of vitamin D assessment in all MS patients especially early after diagnosis and in first demyelinating event (agreement score: 4.9).

The normal range of 25(OH)D have been revised in recent years. Normality is currently between 30 and 100 ng/ml (75 and 250 nmol/l) [24]. Less than 10 ng/ml is considered as deficiency and a range between 11 and 30 ng/ml considered as insufficiency [24, 25]. However for many of the non-classic, extra-bone effect of vitamin D including MS prevention, 40 ng/ml (100 nmol/l) was suggested [24, 26, 27]. Previous studies recommended a threshold of 30 ng/ml for patients with hyperparathyroidism and renal disease stage 3–5 (including dialysis patients) [26].

### Who should be supplemented, which supplement and which dose should be used?

A daily intake of 1000 IU vitamin D resulted in an approximately 10 ng/ml increases in 25(OH)D [28]. However, there are variations in individual response [28]. To reach a serum level of 30 ng/ml, a daily intake of 1000–4000 IU (average ~ 2000 IU) is required [26]. Most studies denoted that even a daily dose of 10,000 IU for several months is not resulted in adverse effects [28]. Sun bathing also could provide vitamin D dose equivalent to an oral consumption of up to 20,000 IU per day. However, in healthy individual who spend long times in sunny environment, serum level of 25(OH)D rarely exceed 100 ng/ml [26].

Although daily vitamin D is considered to be more physiologic, different studies reported that intermittently administered vitamin D has approximately equal effects on 25(OH)D level as cumulative daily dose [29]. A randomized controlled trial (RCT) performed by Ish-shalom reported adverse outcomes with one annual dose of 500,000 IU. According to previous studies daily, weekly, biweekly or monthly strategies are preferred [29]. The panel agreed on daily (agreement score: 3.3), weekly (agreement score: 3.3), biweekly (agreement score: 3.75) or monthly (agreement score: 4.1) strategies. A majority of experts believe that biweekly or monthly strategies resulted in better compliance.

As RCTs did not support the therapeutic effect of high-dose vitamin D, the panel recommended the currently prescribed doses of vitamin D in clinical practice to treat hypovitaminosis and prevent deficiency to MS patients [30]. Thus, in patients with vitamin D insufficiency or deficiency, a large replacing dose was proposed in initial phase (e.g. 50,000 IU pearl of vitamin D per week for 8–12 week [30]) (agreement score: 4.8). Checking the serum vitamin D level and patients’ compliance is recommended after initial phase (agreement score: 4.8).If the level of vitamin D does not reach normal level by 12 weeks, repeting the replacement phase for another 8–12 weeks is recommended (agreement score: 4.8) [30]. According to previous evidences, to maintain the 25(OH)D level above 30 ng/ml, 1500–2000 IU vitamin D/d is required [24]. The specialists suggested 2000 IU/d or equivalent intermittent (weekly, biweekly or monthly) Dose (agreement score: 4.9). The panel was disagreed with increasing the current recommended dose, before the release of the results of the ongoing large RCTs with agreement score of 5.

Up to the best of our knowledge, no high power trial compare vitamin D injection with oral therapy in MS patients. The results of the trials about intramascular vitamin D administration in Non-MS Patients are conflicting. A number of trials reported intra muscular to be more effective [31, 32], and some considered that oral supplementation as superior [33]. Further clinical trials are required to elucidate differences.

Bhargava et al. reported that MS patients have reduces serologic response to vitamin D supplementation. The results of their study proposed the idea that vitamin D pharmacokinetic may differ in MS patients compared to healthy subjects [34]. There is no drug interaction between disease modifying medications used for MS treatment and vitamin D [35].

Daily consumption of vitamin D2 and vitamin D3 seems to have almost similar effect on serum 25(OH)D level. When D3 is available, supplementation with D3 is preferred as it avoids problems with differences in 25(OH)D assay specificity [26] (agreement score: 4.6). More ever, when using intermittent regimen, D3 maintains serum 25(OH)D level consistent for a longer time.

More ever, vitamin D and calcium seemed to act together, such as in the pathogenesis of breast and colorectal cancer, oesteoprosis and probably autoimmune diseases [36]. Cantorna et al., reported that reduced lymphocyte count in the lymph nodes and enhanced IL-4 mRNA in response to 1,25(OH)_2_D administration was occurred solely when calcium intake was sufficient [37]. In a study by Soliu Hänninen, MS patients have hypocalcemia [38]. More ever, proinflammatory cytokines such as IL1-α, IFN-γ and TNF-α which considered pathogenic in MS, also inhibits bone formation and stimulates bone resorption [38] Therefore, the panel recommended a combination of vitamin D and calcium (agreement score:2.8).

### How often should the test be performed?

With daily or weekly dose, at least 3 months of supplementation is needed to reach a plateau [26, 39]. Thus, measurement of 25(OH)D is recommended after 3 months of therapy (agreement score: 4.9). In case of resolving the insufficiency and deficiency, the panel recommended routine check of serum vitamin D level at least two times a year especially the beginning of spring and autumn (agreement score: 4.9).

The panel also recommends monitoring of calcium level at baseline and after 3 months of supplementation in deficient patients (agreement score: 4.9).

Therefore, the panel recommended that further monitoring of PTH, and urinary and serum calcium be performed according to physician decision (agreement score: 4.9).

### Is checking the vitamin D level and supplementation suggested for the family members of MS patients?

As the most of the people does not meet the minimum requirement of Ultraviolet B (UVB), vitamin D supplementation is recommended for the majority of general population. The risk of MS is only 0.2–0.4 % in first degree relatives. However, vitamin D supplementation is inexpensive and safe and also seems to be reasonable even from the general preventive medical point of view alone. However the panel did not reached the consensus about vitamin D level check for all family members of MS Patients. But the panel suggests serum vitamin D evaluation for first degree relatives of MS patients who are at high risk age. If serum level is <40 ng/ml, the panel recommended the currently prescribed doses of vitamin D in clinical practice (50,000 IU/week for 8–12 weeks) to treat hypovitaminosis [30]. The panel recommended the currently suggested maintenance dose of vitamin D (1000 IU/d for <18 year and 1500–2000 IU for adults) to maintain serum level of 25(OH) D above 30 ng/ml for the family memebers of MS patients [24] (agreement score: 4.2).

### In case of pregnancy, should a patient with MS continue vitamin D supplementation and how often it should be checked?

The vitamin D deficiency is highly prevalent in pregnant women worldwide [40, 41]. Results of a study showed that vitamin D level is significantly lower among pregnant MS patients compared to healthy controls [42]. Vitamin D deficiency might increase the risk of preeclampsia [43], bacterial vaginitis [44], low birth weight babies, gestational diabetes, obstructed labor, preterm delivery and miscarriage [45]. In 2011, the Institution of medicine (IOM) published new DRIs of vitamin D for pregnant women that increase from 400 to 600 IU (same as non-pregnant women). However, the new IOM report was based on the skeletal effect of vitamin D and not on other non-classical actions of vitamin D. The 2011 IOM increased the upper limit of vitamin D intake from 2000 to 4000 IU/d. The endocrine society recommended at least 600 IU/d for pregnant women and recommended that for pregnant individuals who are at increasing risk of deficiency, 1500–2000 IU might be needed to maintain vitamin D level above 30 ng/ml. Different studies evaluated the effective dose of vitamin D supplementation during pregnancy. The dose of vitamin D supplement varied widely in different studies (400–4000 IU per day or equivalent single dose). The minimum effective dose which improve pregnancy outcome, but not induce toxicity is still unclear [45]. Also no significant association between vitamin D level and postpartum relapse rate was observed [42]. The panel suggests vitamin D level check and supplementation in case of deficiency/insufficiency before becoming pregnant (agreements score: 4.75). During pregnancy, the panel suggested a daily dose of 1500–2000 IU or equivalent biweekly intake in second and third trimesters (agreement score: 4.75). The panel also suggested 25(OH)D check every 3 months (agreement score: 4.75). In case of serum levels greater than 100 ng/ml, the supplementation should be ceased (agreement score: 5).

### What are the signs and symptoms of vitamin D toxicity and how it should be managed in MS patients?

Like other essential micronutrients, vitamin D intake has a U curve regarding adverse effects due to deficiency and toxicity. Vitamin D toxicity from dietary source is unusual. However, signs of toxicity might appear as a result of high dose vitamin D supplementation [45].

According to endocrine society guideline maintenance dose of <4000 IU/day does not resulted in any notable adverse effects [24]. Consumption of maintenance dose of ≥4000 IU per day might increase the likelihood of hypercalcemia [46–48]. According to the results of Burton et al. study, large replacing dose for short duration did not result in hypercalcemia. As shown in Table 1 they prescribed 40,000 IU/d for 28 weeks to MS patients [49]. Also studies recommended 50,000 IU vitamin D/week for 8–12 to treat hypovitaminosis [30]. Only annual large dose exceeding 300,000 IU vitamin D2 was reported to be associated with an increased risk of hip/femur/wrist fracture in elderly women [48] and greater number of falls [49].

The sign and symptoms of vitamin D intoxication might include: anorexia, nausea and vomiting, abdominal pain, constipation, dehydration, polyuria, polydipsia, nephrocalcinosis, nephrolithiasis, chronic interstitial nephritis, nephrogenic diabetes insipidus and chronic renal failure, paresthesia, hypotonia, seizure, confusion, apathy, coma, hypertension, arrhythmia, bradycardia, cardiomyopathy, calcification, muscle weakness, conjunctival calcification and osteoporosis. The signs of toxicity mostly observed when very large single dose was used. For example IOM reported that >50–60 ng/ml should raise concerns about possible adverse effects (Tolerable Upper Intake Levels: Calcium and Vitamin D). In the “endocrine society guidline” and “practical guideline for central Europe”, >100 ng/ml was considered as the intoxicated level [24, 27]. The panel was agreed with the safety serum level of 100 ng/ml (agreement score: 5). The panel suggest the discontinuation of vitamin D supplementation if the circulating level is above 100 ng/ml and recheck after 6 months regarding vitamin D reserve (agreement score: 5). If the blood level is more than 150 ng/ml, the panel recommended urinalysis because of the risk of hypercalcuria and renal stones [50].

### Dose the amount of vitamin D supplementation should be adjusted according to physical activity level?

RCTs have previously revealed that vitamin D supplementation can significantly improve muscle function and physical performance in vitamin D-deficit individuals [51]. Low 25(OH)D level might indirectly affect physical performance by inducing hyperparathyroidism. PTH has been shown to stimulate IL-6 secretion. IL-6 increment is associated with poor physical function and reduced muscle strength [51].

Previous RCTs recommended 1000 IU vitamin D/day in older individuals with low physical performance [52]. The panel suggests 1000 IU or its intermittent equivalents in MS patients with limited physical activity (agreement score: 4.75).

### Is checking the vitamin D level and supplementation suggested for patients with clinically isolated syndrome?

CIS is defined as first demyelinating event indicating high risk for MS (i.e., one clinical event involving the spinal cord, the optic nerve, the brainstem or cerebellum or occasionally the hemispheres) and at least 2 silent T2 bright areas on a brain or spinal cord MRI (at least one must be in the brain). Previous studies reported that severe vitamin D deficiency is more common among clinically isolated syndrome patients [53]. Also, according to previous studies low serum vitamin D is correlates with increased MS risk in CIS patients [54]. On the other hand, in CIS patients, higher total cholesterol level was associated with an increment in the number of contrast enhancing lesions on brain-MRI and consequently the first clinical event. Brown et al. conducted a study on the relationship between vitamin D metabolits’ level and serum lipoprotein status. They reported that a higher 25(OH)D3 level was associated with higher HDL biomarkers (HDL-c, Apo AII, Apo AI, arylesterase and paroxanase activity) and LDL biomarkers (LDL-c and Apo B). Though, the relationship between HDL-biomarkers and 25(OH)D3 was stronger. Therefore, they proposed that the effect of hypovitaminosis D on lipid profile in CIS patients, might be one of the susceptible cause of its adverse effect on CIS course of disease [55]. The panel suggests vitamin D level check and 8–12 weeks of supplementation in case of insufficiency and deficiency (25(OH)D below 40 ng/ml) for all CIS patients (agreement score: 4).

## Conclusions

Considering the mounting evidences presented here, the consensus recommends:

1. Vitamin D assessment for all MS patients, especially after diagnosis and in the first demyelinating attack.
2. Vitamin D supplementation in case of 25(OH)D level below 40 ng/ml.
3. In patients with vitamin D insufficiency or deficiency, a large replacing dose is proposed in initial phase (e.g. 50,000 IU capsules of D per week for 8–12 week).
4. Checking the serum vitamin D level and patients compliance after initial phase. If the level of vitamin D does not reach normal level by repeating the replacing period for another 8–12 weeks is recommended.
5. Checking serum calcium level at base line and after replacing dose (3 months).
6. A maintenance treatment of 1500–2000 IU daily or equivalent intermittent (weekly, biweekly or monthly) Dose. Biweekly or monthly dose might have better compliance.
7. When D3 is available, supplementation with D3 is preferred.
8. Routine check of serum vitamin D level at least two times a year especially at the beginning of spring and autumn is advised for all treated and untreated patients.
9. Serum vitamin D evaluation for first degree relatives of MS patients at high risk age. Supplementation is recommended in case of insufficiency (25(OH)D less than 40 ng/ml). The panel recommends maintenance dose for family members with normal vitamin D level.
10. The panel suggests the correction of vitamin D deficiency and insufficiency before pregnancy. During pregnancy, the panel suggested a daily dose of 1500–2000 IU or equivalent biweekly intake in 2nd and 3rd trimesters. The panel also suggested 25(OH)D check every 3 months. In case of serum levels greater than 100 ng/ml, the supplementation should be ceased.
11. The panel suggests 1000 IU/d or its intermittent equivalents in MS patients with limited physical activity.
12. The panel suggests vitamin D level check and 8–12 weeks of supplementation in case of serum 25(OH)D level below 40 ng/ml for all CIS patients.

The data of national comprehensive study on household food consumption pattern and nutritional status, Iran, 2001–2003 revealed that average per capita consumption of dairy products in Iran was 0.1–0.18 of western societies (139 g/d compared to 769–1369 g/d) [56]. Also Iranians have limited access to oily fishes as one of the main sources of vitamin D [57]. Considering low intake and high prevalence of vitamin D deficiency, it seems plausible to recommend government and authorities for starting national surveys on vitamin D fortification or supplementation.

More researches are needed to prepare a detailed guideline for vitamin D supplementation as adjuvant therapy in different stages of MS. Our meeting is an attempt to help physicians for better management of vitamin D insufficiency and deficiency, as well as to pave the ground for more researches in this field.

### Ethics approval and consent to participate

The study was approved by local ethical committee of Multiple Sclerosis Research Center (IR.MSRC.rec.1393.324, March 2014).

## Consent for publication

Not applicable.

## Abbreviations

CIS: clinically isolated syndrome
COX-2: cyclo-oxygenase-2
DBP: vitamin D bounding protein
EAE: experimental autoimmune encephalomyelitis
EDSS: expanded disability status scale
IFN-γ: interferon-gamma
IL-10: interleukin-10
IL-12: interleukin-12
IL-17: interleukin-17
IL-6: interleukin-6
IOM: Institution of Medicine
JAK: Janus kinase
MS: multiple sclerosis
MSSS: multiple sclerosis severity scale
NFқB: nuclear factor kB
RCT: randomized controlled trial
RRMS: relapsing-remitting multiple sclerosis
STAT: signal transducer and activator of transcription
TYK: tyrosine kinase
UVB: ultraviolet B
VDR: vitamin D receptor
